# Possible benefits of tomato juice consumption: a pilot study on irradiated human lymphocytes from healthy donors

**DOI:** 10.1186/s12937-017-0248-3

**Published:** 2017-05-12

**Authors:** Ayumi Nakamura, Chieko Itaki, Ayako Saito, Toko Yonezawa, Koichi Aizawa, Ayumi Hirai, Hiroyuki Suganuma, Tomisato Miura, Yasushi Mariya, Siamak Haghdoost

**Affiliations:** 1 Department of Radiological Life Sciences, Division of Medical Life Sciences, Hirosaki University Graduate School of Health Sciences, Aomori, 036-8564 Japan; 2 Department of Disability and Health, Division of Medical Life Sciences, Hirosaki University Graduate School of Health Sciences, Aomori, 036-8564 Japan; 3 Department of Clinical Laboratory Medicine, Hirosaki Central Hospital, Aomori, 036-8188 Japan; 4 Nature and Wellness Research Department, Innovation Division, KAGOME CO., LTD., Tochigi, 329-2762 Japan; 5 Department of Pathologic Analysis, Division of Medical Life Sciences, Hirosaki University Graduate School of Health Sciences, Aomori, 036-8564 Japan; 6 Center for Radiation Protection Research, Department of Molecular Biosciences, The Wenner-Gren Institute, Stockholm University, Stockholm, SE-106 91 Sweden

**Keywords:** Reactive oxygen species, Tomato juice, Lycopene, β-carotene, Radioprotective effect, Human lymphocytes

## Abstract

**Background:**

Reactive oxygen species (ROS) mediate much of the DNA damage caused by ionizing radiation. Among carotenoids, lycopene and β-carotene, present in tomato juice, are known to be strong radical scavengers. The aim of the study was to investigate the effect of tomato juice intake on the levels of DNA damage and oxidative stress in human whole blood induced by in vitro exposure to X-rays.

**Methods:**

Ten healthy adults were asked to drink 190 g of tomato juice, containing 17 mg lycopene and 0.25 mg β-carotene, per day for 3 weeks and then refrain from drinking it for 3 weeks. Peripheral whole blood samples were collected before and after the intake period of tomato juice and after the washout period. The blood samples were exposed in vitro to X-ray doses of 0, 0.1, 0.5, and 2 Gy. Cytogenetic damage was measured using the cytokinesis-block micronucleus (CBMN) assay and the dicentrics (DIC) assay. The level of oxidative stress was determined using serum 8-oxo-7, 8-dihydro-2-deoxyguanosine (8-oxo-dG) and plasma reactive oxygen metabolite-derived compounds (d-ROMs). The concentration of carotenoids in plasma was measured at the three time points.

**Results:**

The levels of 8-oxo-dG tended to decrease during the intake period and increase during the washout period. A non-significant inverse correlation was noted between the plasma concentration of lycopene plus β-carotene and the level of 8-oxo-dG (*P* = 0.064). The radiation-induced MN and DIC frequencies increased in a dose-dependent manner, and when compared at the same dose, the MN and DIC frequencies decreased during the intake period compared with those at baseline and then increased during the washout period. The results suggest that continuous tomato juice consumption non-significantly decreases extracellular 8-oxo-dG, d-ROMs, and MN. Tomato juice intake had minimal or no effect on radiation-induced 8-oxo-dG and d-ROMs. For most radiation doses, continuously tomato juice intake lowered the levels of MN and DIC.

**Conclusion:**

Tomato juice consumption may suppress human lymphocyte DNA damage caused by radiation, but further examination is required.

**Trial registration:**

2014-001 and 2014-R06.

## Background

Oxidative stress is generated by reactive compounds such as reactive oxygen species (ROS) and reactive nitrogen species. ROS react with biomolecules in vivo, resulting in lipid peroxidation, DNA damage, and protein modification. ROS are produced by a variety of processes such as exposure to radiation, alcohol consumption, smoking and excessive exercise. ROS are also produced during cellular metabolism and give rise to DNA damage and protein/lipid modifications. An excessive level of ROS may contribute to the development of various diseases and promotion of the aging process [1–3].

In the present study, we used X-rays to induce DNA damages. X-rays cause DNA damage directly by inducing ionization and excitation of DNA molecules and indirectly through the formation of ROS by radiolysis of cellular water molecules. The indirect action is responsible for approximately 70% of the DNA damage caused by low linear energy transfer ionizing radiation, such as X-rays. In addition to the level of ROS formed by the radiolysis of cellular water, ionizing radiation also increases the endogenous production of free radicals from e.g., mitochondria [4, 5]. Although different DNA repair systems act on the damaged DNA to restore the original DNA sequences, the repair processes are not error free and may result in mutations including deletions, point mutations, and chromosomal aberrations. The aberrations can be measured in the form of dicentrics (DIC) formed as result of mis-repaired double strand breaks [6].

In addition to the DNA molecule, ROS can also react with free DNA precursors in the cytoplasm (nucleotide pool, dNTPs) and modify their structures. Modified dNTPs can be incorporated into the DNA during replication, causing point mutations [7]. One of the most studied modifications of dNTP is 8-hydroxy-2′-deoxyguanosine-triphosphate (8-oxo-dGTP), which is formed when ROS react with deoxyguanosine triphosphate (dGTP). A protein called MTH1 dephosphorylates 8-oxo-dGTP to 8-hydroxy-2′-deoxyguanosine-monophosphate (8-oxo-dGMP). 8-oxo-dGMP will be further dephosphorylated and released from the cells as 8-oxo-7, 8-dihydro-2′-deoxyguanosine (8-oxo-dG) [4]. When endogenous antioxidant defenses are insufficient to reduce the elevated level of ROS, appropriate antioxidant interventions can inhibit, at least partly, the reaction of ROS with biomolecules, particularly DNA, thereby offering protection [8, 9].

Carotenoids, especially lycopene and β-carotene, are found primarily in red and yellow/orange fruits and vegetables such as tomato and carrots. Recent epidemiological studies indicate that the intake of carotenoid-rich foods is associated with reduced risks of chronic diseases, cancer and heart diseases. Tomato-based food products such as tomato juice are important and convenient sources of carotenoids. Tomato juice, in particular, is rich in lycopene and β-carotene. These carotenoids have strong abilities to quench singlet oxygen [10, 11]. Previously, we reported that tomato juice intake decreases oxidative stress in individuals performing extensive physical activity [12]. It was also reported that daily tomato juice consumption for 4 weeks significantly increased the levels of serum lycopene and β-carotene [13]. Therefore, we assumed that the active ingredients in tomato juice suppress excess ROS and eventually provide an antigenotoxic effect. In the present study, we investigated the effect of 3 weeks of tomato juice intake, corresponding to a daily intake of 17 mg of lycopene and 0.25 mg of β-carotene, on the levels of carotenoids and biomarkers of oxidative stress and chromosomal damage in samples of whole blood and after in vitro exposure of the samples to X-rays.

## Methods

### Subjects and blood sampling

Ten healthy donors (5 males and 5 females) aged 22–26 years (mean 23 years) were recruited and asked to drink 190 g of commercially available tomato juice [‘Kagome Tomato Juice (salt added)’, KAGOME CO., LTD., Nagoya, Japan] per day for 3 weeks and then to refrain from drinking it for 3 weeks. The tomato juice was prepared as per industrial standard processes and commercialized in cans. The donors were non-vegetarians, non-smokers and not pregnant. To avoid confounders that could affect the results, the donors were asked to avoid excessive physical training, alcohol consumption, intake of any vitamin supplements and exposure to radiation for diagnostic or treatment purposes during the experimental period.

The nutrition and carotenoid content of the tomato juice used in the study is presented in Table 1. Peripheral blood samples were collected before and after the intake period of tomato juice and following the washout period. Forty mL whole blood was collected at each occasion and divided in 8 tubes; 4 CPT tubes with heparin (BD Biosciences, San Jose, CA, USA) and 4 tubes without anticoagulant (BD Biosciences, Franklin Lakes, NJ, USA). This study was performed in accordance with the ethical standards and was approved by the Ethics Committee of the Hirosaki University Graduate School of Health Sciences (approval number: 2014–001) and the Ethics Committee of KAGOME CO., LTD. (receipt number: 2014-R06).

**Table 1:** The nutrition facts and carotenoid content in the tomato juice

(A)		(B)	
Nutrition facts^a^		Carotenoid content^b^	
Energy (kcal)	38.0	Lutein (mg)	0.127 ± 0.0006
Protein (g)	1.6	β-Cryptoxanthin (mg)	0.042 ± 0.0035
Fat (g)	0.0	α-Carotene (mg)	0.074 ± 0.0003
Carbohydrate (g)	7.2	β-carotene (mg)	0.255 ± 0.0009
Dietary fiber (g)	1.3	Lycopene (mg)	16.9 ± 0.2
Sodium (mg)	210.0		
Sodium chloride equivalent (g)	0.5		
Calcium (mg)	13.0		
Potassium (mg)	530.0		
Sucrose (g)	0.0		

(A) The nutrition facts (B) Carotenoid content in the tomato juice used in this experiment (190 g)

^a^Nutrition facts are according to the product label

^b^Carotenoid content is measured using HPLC (see Method section). Values are given as mean ± SE of three samples

### In vitro X-ray irradiation

Four heparin tubes and four tubes without anticoagulant with 5 mL whole blood in each were cooled and irradiated on ice. The irradiation (150 kVp, 20 mA, 0.5 mm aluminum, and 0.3 mm copper filters) was performed using an X-ray generator (MBR-1520R; Hitachi Medical Co. Ltd., Tokyo, Japan) allowing a 45 cm distance between the beam focus and the samples. The doses were 0, 0.1, 0.5 and 2 Gy, delivered at a dose rate of 1 Gy/min and monitored with a thimble ionization chamber placed next to a sample.

### Isolation of plasma, lymphocytes, and serum from whole blood

Following exposure the irradiated blood samples and control samples were incubated at 37 °C in a water bath for 1 h. Plasma was collected for measuring the concentration of carotenoids, and reactive oxygen metabolites-derived compounds (d-ROMs) and serum was collected for determination of 8-oxo-dG. Mononuclear cells for the cytokinesis-block micronucleus (CBMN) assay and DIC assay were isolated with heparin using CPT tubes according to the provider’s protocol.

### Quantification of carotenoids

Plasma samples of 200 μL were mixed with 10 μL 8-apocarotenal in ethanol [0.0001% (w/v), (Sigma-Aldrich, St. Luis, MO, USA)] as internal standard, 1 mL ethanol containing 0.01% (w/v) butylhydroxytoluene, and 4 mL hexane:dichloromethane [4:1 (v/v)]. The extraction process was repeated if the plasma samples contained precipitated material. After stirring and centrifugation, the supernatants rich in carotenoids were collected and kept in liquid nitrogen. Subsequently, the samples were evaporated to dryness and the residues were re-dissolved in 200 μL hexane:acetone:ethanol:toluene [10:7:6:7 (v/v/v/v)] solution and filtered through a 0.22-μm filter for high-performance liquid chromatography (HPLC) analysis. A C30 carotenoid column [S5 μm, 250 × 2.0 mm I.D. (YMC, Wilmington, NC, USA)] was used at 30 °C, and the SPD-M10 *vp* diode array detector (Shimadzu, Kyoto, Japan) was set at 460 nm. Carotenoids {[lutein, zeaxanthin, β-cryptoxanthin, α-carotene, β-carotene, and lycopene (cis- and trans-isomers)], Extrasynthese, Genay, France} were quantified by determining peak areas in the HPLC chromatograms, calibrated against known standards.

### Measurement of 8-oxo-dG

After complete coagulation of the blood samples, the blood serum was isolated. The 8-oxo-dG concentration of blood serum was measured using enzyme-linked immunosorbent assay (ELISA) as described previously [4, 14]. The ELISA kit was provided by Health Biomarkers Sweden AB (Stockholm, Sweden). Prior to ELISA, 800 μL of blood serum was filtered through a C18 solid phase extraction column (Varian, Lake Forest, CA, USA) according to a previously published method [14]. This step is necessary to remove products other than 8-oxo-dG which could cross-react with the monoclonal antibody used in the kit. A standard curve for 8-oxo-dG (0.01–10 ng/mL) was established for each plate covering the range of 8-oxo-dG in the samples. Validation of the modified ELISA method was performed by HPLC-EC (r^2^ = 0.87, *P* < 0.05) [4]. Comparisons between the ELISA and the HPLC-EC methods showed a linear correlation in the concentration range found in human blood serum [4].

### Measurement of d-ROMs

The plasma concentration of d-ROMs was measured by a 7180 Series Automatic Biochemistry Analyzer (Hitachi, Ltd., Tokyo, Japan). In the d-ROMs test (kit from Diacron International, Grosseto, Italy), the release of the reactive oxygen metabolites (hydroperoxides primarily) and iron (by an acidic buffer) from plasma generates alkoxyl and peroxyl radicals, according to Fenton’s reaction. Such radicals, in turn, oxidize an alkyl-substituted aromatic amine (N, N-dietylparaphenylendiamine) which is included in the kit, thus producing a pink-colored derivative which is photometrically quantified at 505 nm. The concentration of d-ROMs is directly associated with the color intensity and is expressed as Carratelli Units (1 CARR U corresponds to 0.08 mg hydrogen peroxide/dL).

### Cytokinesis-block micronucleus assay

The isolated lymphocytes were cultured in RPMI 1640 medium (Life Technologies, Carlsbad, CA, USA) containing 20% fetal bovine serum (FBS; Japan Bio Serum, Hiroshima, Japan), 1% penicillin/streptomycin (Life Technologies, Carlsbad, CA, USA), and 2% phytohaemagglutinin-HA15 (Remel, Lenexa, KS, USA) in a 15 mL Falcon tube. The cells were incubated in a humidified incubator at 37 °C with 5% CO_2_ for exactly 44 h. Following the incubation, the cells were treated with Cytochalasin-B (Wako Pure Chemical Industries, Ltd., Osaka, Japan) at a final concentration of 6 μg/mL and then incubated for further 28 h in the cell culture incubator. Following the incubation, the cells were washed and suspended in RPMI 1640 medium containing 2% FBS. Further, 300 μL of cell suspension was spun onto a slide glass using Cytospin™ 4 Cytocentrifuge (Thermo Fisher Scientific, Inc., Waltham, MA, USA). The cells were fixed on the slides and stained with Diff-Quik (Sysmex, Kobe, Japan). The slides were scored at 400× magnification using a bright-field microscope (BX61: Olympus, Tokyo, Japan). At least 1000 binucleated cells (BNCs) were scored per slide and the results were expressed as number of MN/1000 BNCs.

### Dicentric assay

The isolated lymphocytes were cultured in RPMI 1640 medium supplemented with 20% FBS, 1% penicillin/streptomycin, and 2% phytohaemagglutinin-HA15, and 0.05 μg/mL colcemid (Life Technologies, Carlsbad, CA, USA) in a 15 mL Falcon tube and incubated in a humidified incubator at 37 °C with 5% CO_2_. Following 48 h incubation, cells were harvested, treated with 0.075 M KCl, and fixed with cold fixative (methanol: acetic acid 3:1 (v/v)). Following centrifugation, the cell pellets were re-suspended in 1–2 mL of fixative. A temperature-humidity-controlled chamber (Hanabi metaphase spreader, ADSTEC, Japan) was used for spreading of the metaphases on pre-cleaned slide glass (Matsunami Glass Ind., Japan). The slides were then stained with Giemsa solution and mounted with a cover glass. The metaphase cells were found in the AutoCapt mode using two sets of AXIOImager Z.2 microscopes (Carl Zeiss AG, Oberkochen, Germany) equipped with CCD cameras and Metafer 4 software (MetaSystem GmbH, Altlusshein, Germany). Metaphases for scoring were selected in manual mode. Using the selected metaphase images, all DIC in 500 metaphases were scored. Data were presented as the number of DIC/500 metaphases. The cells were analyzed according to the criteria described by the International Atomic Energy Agency in 2011.

### Statistical analysis

Statistical analyses were performed using the Excel 2013 software (Microsoft Corp., Redmond, WA, USA) equipped with the Statcel 3 add-in software package. Data were expressed as mean ± standard error (SE). Statistical analyses were conducted by paired *t* test, Wilcoxon signed-ranks test, Bonferroni/Dunn test, and simple regression analysis. The linear regression analysis was performed to investigate the correlation between the plasma lycopene/β-carotene concentrations and studied biomarkers. *P* values of less than 0.05 were considered to indicate statistical significance.

## Results

We compared values of each biomarker among three measurements, namely before and after the tomato juice intake and after the following washout period, and the measurements were defined as the “baseline,” “intake,” and “washout”.

### Changes in the concentration of carotenoids in blood

**Fig. 1:**
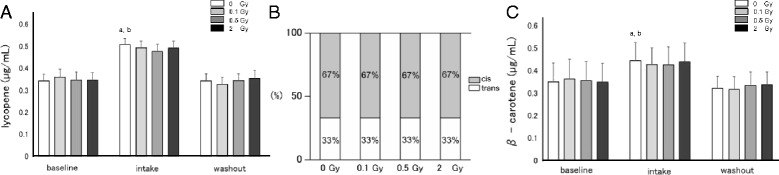
Radiation effects on the level of main carotenoids in human blood. **a** Concentration of lycopene according to radiation dose at each time point. Values are given as mean ± SE of 10 donors. **b** Structure ratio of lycopene (cis/trans) according to radiation dose. The ratio was constant between the different radiation doses. **c** Concentration of β-carotene according to radiation dose at each time point. Values are given as mean ± SE of 10 donors. a: different from baseline *P* < 0.05, b: different from washout *P* < 0.05 by Bonferroni/Dunn test. Concentrations of carotenoids after each period did not differ significantly by radiation (1**a**, 1**c**)

The plasma levels of six major carotenoids at the three different time points are shown in Table 2, and the sum of the concentrations of lycopene and β-carotene in tomato juice was calculated. The plasma concentration of lycopene significantly increased after 3 weeks of tomato juice intake (0.51 ± 0.03 μg/mL) compared with that at the baseline level (0.34 ± 0.03 μg/mL) and reduced to the baseline level after the washout period (0.34 ± 0.03 μg/mL). The concentration of β-carotene also increased from 0.35 ± 0.08 μg/mL to 0.44 ± 0.08 μg/mL due to continual intake of tomato juice and decreased to 0.32 ± 0.05 μg/mL during the washout period. Plasma levels of other carotenoids (lutein, zeaxanthin, β-cryptoxanthin, α-carotene) were not notably changed between the three measurements. Neither the concentration of lycopene nor its cis/trans ratio differed significantly between samples that received different doses of radiation and non-irradiated samples (Fig. 1a and b), indicating that radiation at doses up to 2 Gy do not degrade and/or isomerize lycopene. Also, the concentration of β-carotene in the blood remained largely unchanged by radiation (Fig. 1c).

**Table 2:** The concentration of carotenoids in the human plasma

Carotenoids (μg/mL)	Baseline	Intake	Washout
	Mean ± SE	Mean ± SE	Mean ± SE
Lutein	0.23 ± 0.03	0.23 ± 0.02	0.21 ± 0.02
Zeaxanthin	0.06 ± 0.01	0.05 ± 0.01	0.05 ± 0.01
β-Cryptoxanthin	0.12 ± 0.02	0.13 ± 0.02	0.12 ± 0.01
α-Carotene	0.15 ± 0.02	0.13 ± 0.02	0.12 ± 0.01
β-Carotene	0.35 ± 0.08	0.44 ± 0.08 ^a, b^	0.32 ± 0.05
Lycopene	0.34 ± 0.03	0.51 ± 0.03 ^a, b^	0.34 ± 0.03

Values are given as mean ± SE of 10 donors. ^a^: different from baseline (*P* < 0.05), ^b^: different from washout (*P* < 0.05) by paired *t* test

### Changes in the level of oxidative stress-related markers

**Fig. 2:**
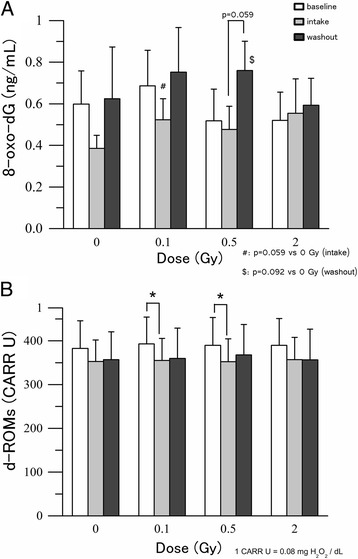
The level of oxidative stress-related markers in human blood. **a** Level of 8-oxo-dG in serum. **b** Level of d-ROMs in plasma. Values are given as mean ± SE of 10 donors. *P < 0.05, #: different between base line and intake period *P* = 0.059, $: different between base line and washout period *P* = 0.092 by (**a**) Wilcoxon signed-ranks test and (**b**) paired *t* test

We assessed the effect of radiation on two oxidative stress-related markers: extracellular 8-oxo-dG (Fig. 2a) in serum and d-ROMs (Fig. 2b) in plasma. In non-irradiated samples, the concentration of extracellular 8-oxo-dG decreased during the intake period (from 0.60 ± 0.16 at baseline to 0.39 ± 0.06 ng/mL) and increased after the washout period (to 0.62 ± 0.25 ng/mL), indicating that tomato juice intake may decrease 8-oxo-dG levels in serum. However, this effect was not significant. The concentrations at baseline, intake and washout in samples exposed to 0.1 and 0.5 Gy showed a similar pattern. At 0.5 Gy the increase of 8-oxo-dG during the washout period was close to significance (*P* = 0.059). However, no protective effect was observed at 2 Gy (Fig. 2a).

Generally, it is accepted that the concentration of d-ROMs reflects oxidative stress, and we observed that the levels of d-ROMs after exposure to 0.1 and 0.5 Gy significantly decreased during the intake period compared with those at baseline (Fig. 2b). The levels of d-ROMs at 2 Gy at baseline, after intake, and after washout showed a similar pattern. However, the differences were not significant.

### Differential analysis of the lycopene plus β-carotene concentration at baseline

**Fig. 3:**
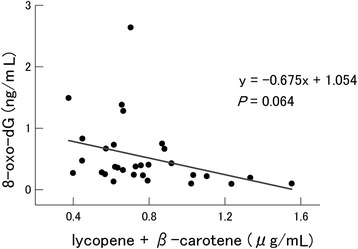
Relationship between concentration of lycopene plus β-carotene and level of 8-oxo-dG in serum in the non-irradiated samples. The *P* value was determined by linear regression analysis

We analyzed correlations of the levels of circulating lycopene and β-carotene, alone or together, with all biomarkers assessed in the study. A slight inverse correlation was found only between the plasma lycopene plus β-carotene concentration and the extracellular 8-oxo-dG level (Fig. 3). This observation indicated that lycopene and β-carotene may reduce the serum levels of 8-oxo-dG (*P* = 0.064). The circulating plasma lycopene plus β-carotene concentrations at baseline in the 10 donors ranged from 0.38 to 1.24 μg/mL. Therefore, the participants were divided into two groups based on their initial plasma lycopene plus β-carotene concentrations (less than or more than 0.635 μg/mL, median value) to evaluate the differences in radiation-induced cytogenetic damage. Group A, in which the plasma lycopene plus β-carotene level exceeded 0.635 μg/mL, consisted of one male and four females, and group B (less than 0.635 μg/mL) consisted of four males and one female. Although significant differences were not observed in cytogenetic damage in Group A, there were some significant differences in Group B (Table 3). For example, the serum levels of 8-oxo-dG were significantly higher following the washout period (1.04 ± 0.21 ng/mL) than immediately after tomato juice intake (0.53 ± 0.12 ng/mL) in samples irradiated with 0.5 Gy in Group B. The number of DIC per 500 metaphases was also significantly lower following the intake (14.99 ± 1.31 ng/mL) than at baseline (18.65 ± 1.86 ng/mL) in samples irradiated with 0.5 Gy. It also increased after the washout period compared with the values immediately following the intake in samples irradiated with 0.5 Gy (from 14.99 ± 1.31 to 20.86 ± 1.96) and 2 Gy (from 85.64 ± 3.26 to 99.73 ± 5.60), but these values were not significant.

**Table 3:** The level of 8-oxo-dG in human serum and cytogenetic damage in human lymphocytes in accordance with lycopene plus β-carotene concentration

		Dose (Gy)	Baseline	Intake	Washout
			Mean ± SE	Mean ± SE	Mean ± SE
8-oxo-dG	(A)	0	0.46 ± 0.23	0.32 ± 0.10	0.28 ± 0.10
(ng/mL)		0.1	0.66 ± 0.30	0.52 ± 0.21	0.74 ± 0.35
		0.5	0.58 ± 0.30	0.42 ± 0.20	0.48 ± 0.09
		2	0.41 ± 0.19	0.34 ± 0.08	0.49 ± 0.17
	(B)	0	0.73 ± 0.22	0.45 ± 0.08	0.97 ± 0.46
		0.1	0.71 ± 0.21	0.53 ± 0.04	0.76 ± 0.29
		0.5	0.45 ± 0.11	0.53 ± 0.12 ^b^	1.04 ± 0.21
		2	0.63 ± 0.20	0.77 ± 0.30	0.69 ± 0.20
MN	(A)	0	22.10 ± 2.96	17.93 ± 4.45	24.07 ± 4.34
(/1000 BNC)		0.1	22.86 ± 1.93	20.54 ± 1.78	28.91 ± 3.59
		0.5	68.98 ± 8.04	54.88 ± 4.87	57.05 ± 8.38
		2	281.6 ± 15.6	330.7 ± 26.7	337.9 ± 19.9
	(B)	0	20.17 ± 2.97	14.30 ± 3.07	24.94 ± 5.63
		0.1	22.43 ± 6.45	20.84 ± 4.32	24.27 ± 3.79
		0.5	57.84 ± 7.61	50.06 ± 4.06	53.01 ± 4.19
		2	286.6 ± 12.2	300.2 ± 26.7	282.6 ± 17.8
DIC	(A)	0	0	0.18 ± 0.18	0
(/500 metaphase)		0.1	0.92 ± 0.49	0.38 ± 0.23	0.78 ± 0.20
		0.5	21.31 ± 1.33	19.93 ± 0.87	21.1 ± 1.71
		2	98.72 ± 6.24	93.47 ± 4.32	105.4 ± 7.1
	(B)	0	0.19 ± 0.19	0	0.19 ± 0.19
		0.1	0.32 ± 0.32	0.58 ± 0.39	0.40 ± 0.24
		0.5	18.65 ± 1.86	14.99 ± 1.31 ^a,#^	20.86 ± 1.96
		2	89.45 ± 6.13	85.64 ± 3.26 ^$^	99.73 ± 5.60

(A) Group of individuals where the lycopene plus β-carotene concentration was higher than the median value at baseline (>0.635 μg/mL). *N* = 5 (1 male, 4 females) (B) Group of individuals where the lycopene plus β-carotene concentration was less than the median value at baseline (<0.635 μg/mL). *N* = 5 (4 males, 1 female) Values are given as mean ± SE of five donors. a: different from the baseline (*P* < 0.05), b: different from the washout period (*P* < 0.05), #: different from the washout (*P* = 0.056), $: different from the washout (*P* = 0.056) by Wilcoxon signed-ranks test (8-oxo-dG) and paired *t* test (MN and DIC)

### Changes in the levels of MN and DIC

**Fig. 4:**
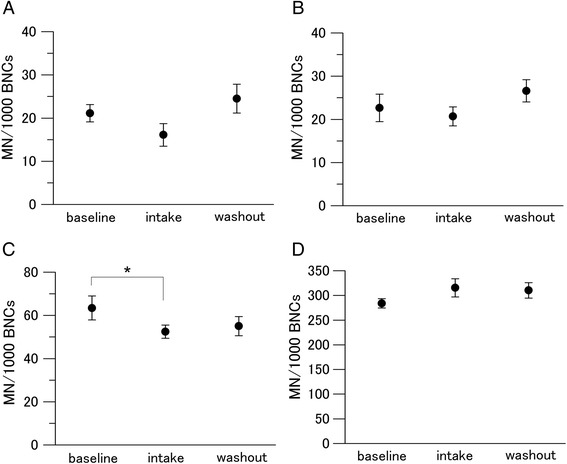
Changes in the level of MN in human lymphocytes. Values are given as mean ± SE of 10 donors. *P < 0.05 by paired *t* test. **a** 0 Gy. **b** 0.1 Gy. **c** 0.5 Gy. **d** 2 Gy

**Fig. 5:**
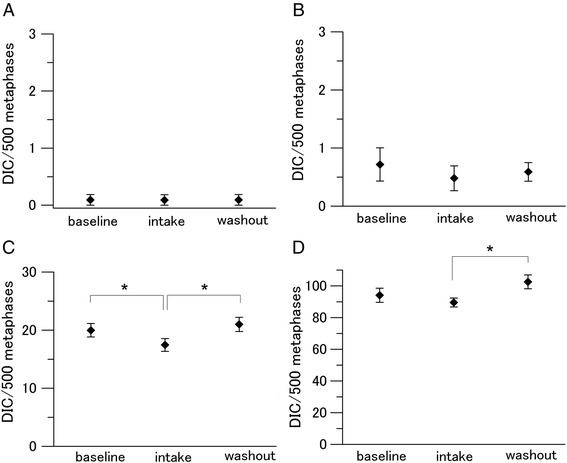
Changes in the level of DIC in human lymphocytes. Values are given as mean ± SE of 10 donors. *P < 0.05 by paired *t* test. **a** 0 Gy. **b** 0.1 Gy. **c** 0.5 Gy. **d** 2 Gy

We measured the MN and DIC frequencies to assess radiation-induced cytogenetic damage in lymphocytes (Figs. 4 and 5). Tomato juice intake decreased the steady-state levels of MN per 1000 BNCs from 21.13 ± 2.00 to 16.11 ± 2.62, albeit not significantly (Fig. 4a). Decreased levels of MN following tomato juice intake were also found in the samples exposed to 0.1 Gy (non-significant effect) and 0.5 Gy (from 63.41 ± 5.54 to 52.47 ± 3.09; significant effect). No effect was found in the samples exposed to 2 Gy, indicating that the capacity of the tomato juice intake was inadequate to protect DNA against MN induction at this high dose. After the washout period, the MN frequencies per 1000 BNCs increased to the baseline levels. On comparing the levels of DIC in non-irradiated baseline samples with samples obtained after tomato juice intake and washout, no notable changes were observed (Fig. 5). The number of DIC per 500 metaphases decreased significantly during the intake period in the samples exposed to 0.5 Gy (from 19.98 ± 1.17 to 17.46 ± 1.11). Furthermore, the number of DIC per 500 metaphases increased significantly after the washout period in the samples exposed to 0.5 Gy (from 17.46 ± 1.11 to 20.99 ± 1.23) and 2 Gy (from 89.56 ± 2.86 to 102.58 ± 4.36).

## Discussion

The present study is based on a small number of healthy donors. Thus, it will be difficult to apply the findings to the general population, and our results should be considered as preliminary. Nevertheless, despite the small number of participants in the present investigation, the results indicate that drinking 190 g of tomato juice every day for 3 weeks decreases the steady state levels of 8-oxo-dG in the blood serum, as well as MN in the blood lymphocytes, although not significantly. No such effects were observed on the levels of d-ROMs. This could be due to the sensitivity of the methods, as extracellular 8-oxo-dG appears to be a more sensitive marker than d-ROMs. We did not observe any differences at the steady-state level of DIC with and without tomato juice intake. This could be due to the fact that DIC is a specific marker of radiation exposure [15]. The overall results indicate that the intake of 190 g of tomato juice per day for 3 weeks may be beneficial for healthy donors.

The baseline MN frequency in the general Japanese population has been reported to be 4–7 per 1000 BNCs [16, 17], which is lower than our observations. This could be due to differences in the protocols and slide preparation methods employed. However, in our previous publication we observed similar levels of MN as in the present investigation [18].

Next, we asked whether tomato juice intake could decrease the levels of radiation-induced oxidative stress biomarkers, namely extracellular 8-oxo-dG and d-ROMs. The reasons for choosing the stress biomarkers are: a) we know the mechanisms of radiation-induced damage in DNA and nucleotide pool [14], b) it is known that low doses of ionizing radiation induce oxidative stress [5], and c) there is worldwide concern regarding the increasing use of radiation for diagnostic purposes, especially in the low-dose region (<0.1 Gy) [19]. If tomato juice could decrease radiation-induced oxidative stress and cytogenetic damage, then it could have beneficial effects for healthy people exposed to low-dose radiation, e.g., due to medical diagnosis. For these reasons, we obtained blood samples from the donors at three time points (baseline, intake, and washout) and exposed them to 0.1, 0.5, or 2 Gy of X-rays. We observed dose-dependent increases in MN and DIC frequencies but not in extracellular 8-oxo-dG and d-ROM levels. In the case of MN and DIC, we observed that tomato juice consumption reduced radiation-induced frequencies of these biomarkers significantly at 0.5 Gy. Drinking tomato juice also resulted in decreased levels of MN and DIC upon exposure to 0.1 Gy, although the results were not statistically significant.

The levels of extracellular 8-oxo-dG in samples exposed to 0.1 and 0.5 Gy were slightly higher than those in the controls, whereas the levels of d-ROMs were unchanged following irradiation. Previously, we have shown that the major pathways that are activated in response to doses below 0.2 Gy are related to oxidative stress [4]. In contrast, most cellular response pathways following exposure to doses above 0.5 Gy are related to DNA repair, cell cycle checkpoint activation and apoptosis, due to extensive DNA damage production. Leukocytes are radiosensitive cells, so when exposed to 0.5 or 2 Gy of radiation, they will likely die through apoptosis or mitotic catastrophe rather than attempt to produce 8-oxo-dG or other stress markers. This could explain why we observed radiation-induced extracellular 8-oxo-dG production in response to 0.1 Gy of radiation. Matos et al. investigated the effect of lycopene treatment on oxidative DNA damage in the livers of male Wistar rats subjected to intraperitoneal ferric nitrilotriacetate administration. Five days of lycopene supplementation (10 mg/kg body weight/day) almost completely prevented the formation of 8-oxo-dG in the DNA of liver cells [20]. Although the applied 8-oxo-dG-forming agent and the investigated type of tissue were different from those employed in this study, these results suggest that lycopene could protect against DNA base oxidation. We also observed that tomato juice intake generally decreases the levels of d-ROMs but radiation has a minimal, if any, effect at the levels of d-ROMs. None of the oxidative stress-related markers revealed a protective effect of tomato juice in response to 2 Gy of radiation, while the analyses of cytogenetic damage disclosed an effect after this dose. In the intake period, reductions in MN frequencies were observed in the samples irradiated with 0.5 Gy, and reductions in DIC frequencies were observed in the samples irradiated with 0.5 and 2 Gy, suggesting that consumption of tomato juice protected against ionizing radiation-induced DNA damage.

It was previously reported that the addition of lycopene to cell cultures could reduce the MN frequency [21, 22] and DIC frequency following exposure to radiation [23]. These findings are consistent with our data suggesting that lycopene can protect against DNA damage [24–26]. Ingestion of tomato extracts has previously been reported to reduce DNA damage, and this property of tomatoes has also been attributed to components other than lycopene [25]. The present study revealed that the ingestion of tomato juice significantly increased the circulating levels of lycopene by 1.5 and β-carotene by 1.26-fold (Table 1). This result is consistent with those of previous reports [27, 28]. In addition, a non-significant inverse correlation was observed between the plasma concentrations of lycopene plus β-carotene and the levels of extracellular 8-oxo-dG (Fig. 3). A weaker and non-significant inverse correlation was observed between the plasma concentrations of lycopene plus β-carotene and the levels of MN (data not shown), whereas no correlations were found for lycopene plus β-carotene levels with d-ROMs or DIC. The results indicate that lycopene together with β-carotene may play an important role in reducing 8-oxo-dG levels. It was reported that β-carotene has radical-scavenging activity and that β-carotene supplementation decreases DNA damages in lymphocytes [29]. However, the positive health effects of β-carotene have not been fully clarified. Although several studies demonstrated that β-carotene–rich foods have positive effects on human health [29], there are indications in the literature that β-carotene supplementation can increase the risk of lung cancer among smokers and/or asbestos-exposed workers [30–32]. One possible mechanism is that β-carotene combined with cigarette smoke alters the regulation of heme oxygenase1 via its transcriptional factor Bach1, which modulates cell proliferation [33]. This aspect was considered in the present investigation, as smokers were excluded. Furthermore, tomatoes contain various antioxidants such as α-tocopherol, vitamin C, and polyphenols. Although our observations in the present study indicated that lycopene and β-carotene are active components that reduce blood oxidative stress levels, we believe that further experiments are needed to clarify the active components in tomatoes.

It has been reported that lycopene is oxidized by the addition of radicals in vitro. We assumed that lycopene thus quenched the ROS induced by radiation. However, radiation doses used in the present investigation did not degrade lycopene and the cis/trans ratio was maintained.

We compared donors with high baseline levels of lycopene and β-carotene with those with low baseline levels. Interestingly, in the former group, tomato juice consumption did not notably influence any of the parameters. However, it significantly reduced 8-oxo-dG and DIC levels in the latter group. These data indicate that it is more beneficial for people with low baseline levels of lycopene and β-carotene to drink tomato juice. Thus, when we tailor this small intervention to healthy people scheduled to undergo CT at a dose of approximately 50 mGy, they may experience the most beneficial effect by consuming tomato juice, as most of the biological effects of low-dose radiation are mediated by elevated endogenous ROS formation [5].

Additionally, healthy non-smokers with high levels of 8-oxo-dG or MN at steady state should also benefit from continuous tomato juice consumption. However, considering the small number of donors and the duration of the study, the obtained results should be considered as preliminary. It is also important to mention that ROS and other reactive species play important roles in normal physiological functions; e.g., ROS are produced by macrophages to kill bacteria and by xenobiotic systems for neutralizing toxins. A recent study demonstrated that antioxidant supplementation reduces ROS levels and DNA damage but increases tumor cell proliferation and tumor growth in mice [34]. The authors also revealed that tumor cells proliferate faster when oxidative stress is suppressed. Their explanation for these results was that a feedback mechanism in lung cancer cells downregulates the endogenous ROS defense system when ROS production is suppressed by antioxidants. However, this study used genetically modified mice that already had lung cancer as well as lung cancer cell lines in culture. The results from genetically modified mice with lung cancer and cancer cells in culture will be difficult to apply to healthy humans or considered in cancer initiation and prevention. This study provides important information regarding the negative effects of Vitamin E and NAC in people with lung cancer or an elevated risk for lung cancer, e.g., smokers. However, in the present study, we aimed to investigate the effects of tomato juice consumption in healthy individuals.

## Conclusion

In conclusion, our results suggest that continuously drinking tomato juice could decrease the steady state levels of extracellular 8-oxo-dG and MN. Generally, tomato juice intake lowered the induction of MN and DIC in exposed cells. Additionally, our results suggest that lycopene and β-carotene are active compounds in tomato juice. The juice also contains other antioxidants, the roles of which were not studied in the present investigation. It is important to mention that the obtained results are based on 10 healthy donors with great individual variation in the results, and the findings should be considered as preliminary and be verified in larger setups.

## Abbreviations

8-oxo-dG: 8-oxo-7, 8-dihydro-2′-deoxyguanosine
8-oxo-dGMP: 8-hydroxy-2′-deoxyguanosine-monophosphate
8-oxo-dGTP: 8-hydroxy-2′-deoxyguanosine-triphosphate
BNCs: Binucleated cells
CBMN: Cytokinesis-block micronucleus
dGTP: Deoxyguanosine triphosphate
DIC: Dicentrics
dNTP: Deoxynucleoside triphosphate
d-ROMs: Reactive oxygen metabolite-derived compounds
ELISA: Enzyme-linked immunosorbent assay
FBS: Fetal bovine serum
HPLC: High-performance liquid chromatography
MN: Micronuclei
ROS: Reactive oxygen species
SE: Standard error
